# Association of Kidney Function With Incident Heart Failure: An Analysis of the Women's Health Initiative

**DOI:** 10.1161/JAHA.124.037051

**Published:** 2025-02-25

**Authors:** Richard K. Cheng, Mary B. Roberts, Nisha Bansal, Kerryn Reding, Taufiq Salahuddin, Mamas Mamas, Michael LaMonte, Aladdin H. Shadyab, Nora Franceschini, Liviu Klein, JoAnn E. Manson, Charles B. Eaton

**Affiliations:** ^1^ Division of Cardiology, Department of Medicine University of Washington Seattle WA USA; ^2^ Department of Radiology University of Washington Seattle WA USA; ^3^ Center for Primary Care and Prevention Kent Hospital Pawtucket RI USA; ^4^ Division of Nephrology, Department of Medicine University of Washington Seattle WA USA; ^5^ Department of Biobehavioral Nursing and Health Informatics, School of Nursing University of Washington Seattle WA USA; ^6^ Division of Cardiology Keele University Keele UK; ^7^ Department of Epidemiology, School of Public Health and Health Professions University at Buffalo – SUNY Buffalo NY USA; ^8^ Herbert Wertheim School of Public Health and Human Longevity Science University of California San Diego, La Jolla CA USA; ^9^ Department of Epidemiology University of North Carolina Chapel Hill NC USA; ^10^ Division of Cardiology, Department of Medicine University of California San Francisco San Francisco CA USA; ^11^ Division of Preventive Medicine Brigham and Women’s Hospital, Harvard Medical School Boston MA USA; ^12^ Department of Family Medicine Warren Alpert Medical School of Brown University Providence RI USA; ^13^ Department of Epidemiology School of Public Health of Brown University Providence RI USA

**Keywords:** chronic kidney disease, heart failure, HFpEF, Heart Failure

## Abstract

**Background:**

Studies have shown an association of chronic kidney disease with heart failure (HF); however, this association has not been adequately examined in postmenopausal women, who are at heightened risk of both chronic kidney disease and HF. Additionally, association with HF subtypes is not well characterized.

**Methods and Results:**

Incident HF was defined as first hospitalization for acute decompensated HF, obtained by self‐reported outcomes followed by physician adjudication through review of hospital records. Chronic kidney disease was defined using estimated glomerular filtration rate (eGFR). Restricted cubic splines tested the association of eGFR with incident overall HF, and HF with reduced ejection fraction (HFrEF) and preserved EF (HFpEF). Cox proportional hazards regression models evaluated the multivariable‐adjusted association of eGFR categories with incident HF and its subtypes. The primary analysis included 23 309 women with 11 814 eGFR ≥90, 10 191 eGFR between 60 and 89, 1048 eGFR between 45 and 59 and 256 eGFR <45 mL/min per 1.73 m^2^. For overall HF, HFrEF and HFpEF, there was a stepwise increase in risk for incident HF with declining eGFR category. Associations were stronger for HFpEF (hazard ratio [HR], 2.80 [95% CI, 2.36–3.32]) than for HFrEF (HR, 2.18 [95% CI, 1.66–2.87]) for eGFR <45 as compared with eGFR ≥90. Heterogeneity of the HF subdistributions (HFpEF versus HFrEF) was significant (*P*=0.017).

**Conclusions:**

Kidney dysfunction is associated with incident HF in postmenopausal women. Although lower eGFR is associated with both incident HFrEF and HFpEF, the association is stronger with HFpEF.

**Registration:**

URL: https://clinicaltrials.gov; Unique Identifier: NCT00000611.

**Nonstandard Abbreviations and Acronyms**
HFmrEF: heart failure with midrange ejection fraction
HFpEF: heart failure with preserved ejection fraction
HFrEF: Heart failure with reduced ejection fraction
WHI: Women's Health Initiative

**Clinical Perspective**

**What Is New?**

- Lower estimated glomerular filtration rate is associated with increased risk of incident heart failure (HF). Our findings are consistent with prior studies demonstrating that chronic kidney disease is associated with risk for incident HF; however, many prior studies did not stratify HF into subtypes.
- Our study adds to existing knowledge by showing that the association of estimated glomerular filtration rate is stronger in HF with preserved ejection fraction than for HF with reduced ejection fraction in women.

**What Are the Clinical Implications?**

- Women with chronic kidney disease should be closely monitored for development of clinical HF.
- Future studies are needed to assess whether targeting mechanisms of HF in individuals with chronic kidney disease may be beneficial.

Cardiovascular disease and chronic kidney disease (CKD) frequently coexist due to shared risk factors such as hypertension, obesity, and insulin resistance, as well as kidney dysfunction related mechanisms of microvascular dysfunction, systemic inflammation, neurohormonal imbalance, and dysregulation of mineral metabolism.^1^, ^2^, ^3^

With the aging population, an increase in multimorbidity will be more frequently encountered, and estimated projections include a growing economic burden and number of deaths due to heart failure (HF).^4^ Similarly, the incidence of CKD increases with advancing age and is associated with accelerated aging of the cardiovascular system.^1^ Additionally, sex‐specific differences exist for both CKD,^5^ with higher prevalence but lower predialysis mortality in women, and for HF,^6^ with a higher proportion of HF with preserved (HFpEF) left ventricular EF (LVEF) in women. Women are underrepresented in both kidney^7^ and cardiovascular clinical trials.^8^

Prior data have shown an association of CKD with prevalent overall HF and for HF with reduced (HFrEF) and HFpEF.^9^ Furthermore, previous studies found that in community‐dwelling older adults in the CHS (Cardiovascular Health Study), CKD with eGFR <45 mL/min per 1.73 m^2^, as estimated by the CKD‐Epidemiology Collaboration (CKD‐EPI) equation, was associated with incident HF.^10^ In the ARIC (Atherosclerosis Risk in Communities) study, estimated glomerular filtration rate (eGFR) <60, as estimated by the MDRD (Modification of Diet in Renal Disease) study equation, was associated with incident HF.^11^ Similarly, an analysis of the pooled cohorts of the Jackson Heart Study, CHS and the Multi‐Ethnic Study of Atherosclerosis found that the absolute risk difference in people with versus without CKD as defined by eGFR <60 was greatest for HF.^12^

However, data are limited on whether the association of kidney function with incident HF is applicable to postmenopausal women, or different for HFrEF versus HFpEF. The WHI (Women's Health Initiative) is well positioned to address these gaps in knowledge as a large prospective study in postmenopausal women with adjudicated events and phenotyping of HF.

In the current study, we sought to determine the association of kidney function as estimated by eGFR using the CKD‐EPI 2021 equation with incident HF in the WHI cohort.

## METHODS

Data and materials from the WHI have been made publicly available at BioLINCC and can be accessed at https://biolincc.nhlbi.nih.gov/studies/whi‐other/. The SAS code and specific variables used for this analysis are available from the corresponding author upon reasonable request. Because of the sensitive nature of the data collected for this study, requests to access additional data from qualified researchers trained in human subject confidentiality protocols may be sent to the WHI Clinical Coordinating Center at helpdesk@whi.org.

### Study Population

The WHI is a nationwide, prospective cohort study of 161 808 generally healthy US postmenopausal women, aged 50 to 79 years, enrolled at 40 clinical centers between 1993 and 1998.^13^, ^14^ Women were enrolled in either 1 or more of 3 clinical trials or an observational study with follow‐up through 2005. At the end of the initial follow‐up, WHI women were asked to participate in subsequent extension studies, with follow‐up currently ongoing. A subgroup of women oversampling for Black and Hispanic women, as well as hormone trial participants, were evaluated for HFrEF and HFpEF using detailed review of medical records including echocardiographic and other imaging data, blood biomarkers, and clinical assessments (N=44 174).

**Figure 1:**
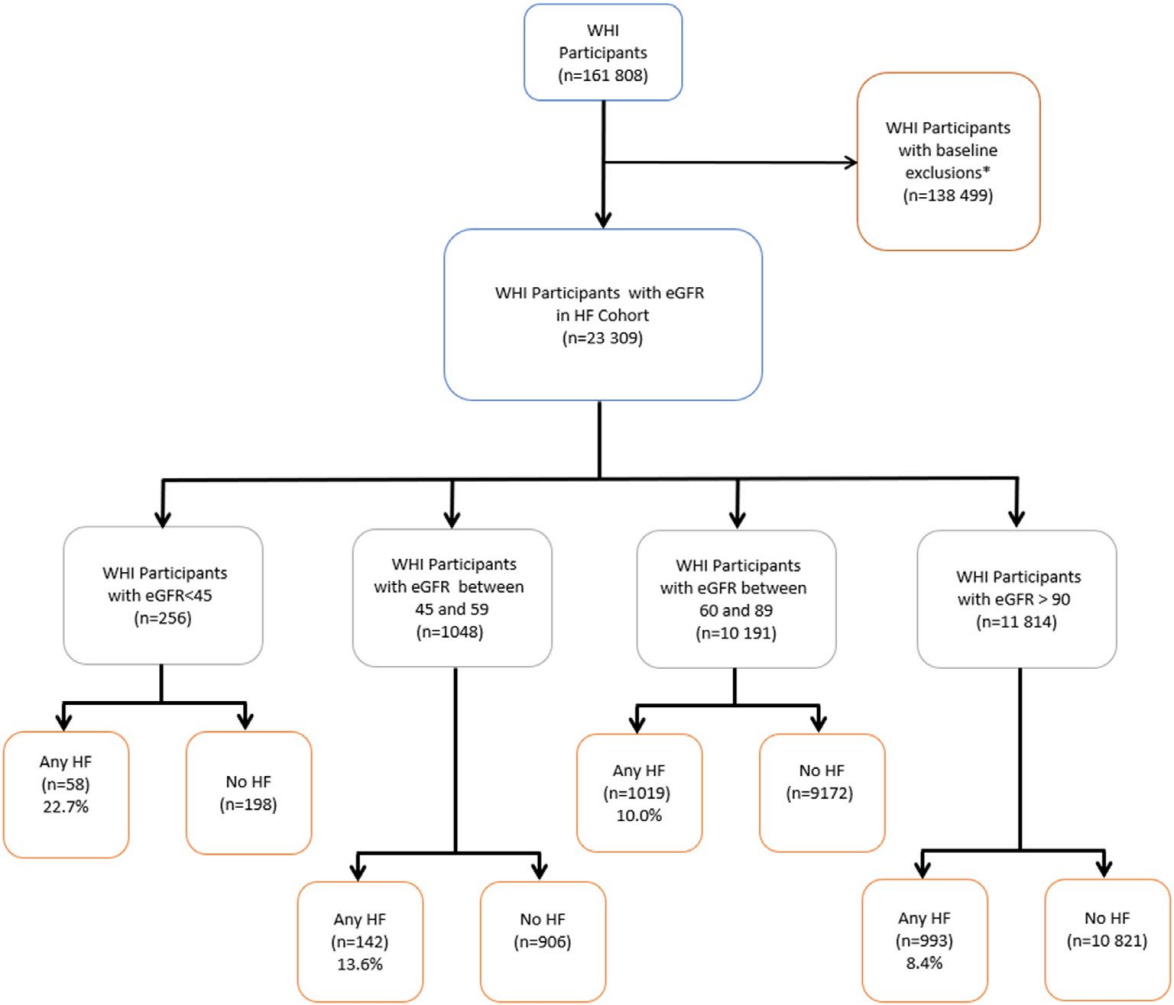
Consolidated Standards of Reporting Trials diagram for the current study. The final cohort included 23 309 participants after applying exclusion criteria. *Exclusion criteria included no creatinine (n, 137 603), preexisting heart failure (n, 423), not part of the heart failure cohort (n, 459) or missing follow‐up (n, 14). eGFR indicates estimated glomerular filtration rate; HF, heart failure; and WHI, Women's Health Initiative.

For the current analysis, participants were included if they had laboratory values available to estimate a baseline (1993–1998) eGFR with the CKD‐EPI 2021 equation using measured serum creatinine. Additionally, participants were excluded if they self‐reported a history of HF at baseline, were not part of the HFrEF/HFpEF subcohort, or were missing follow‐up information. The final study cohort consisted of 23 309 women (Figure 1). Informed consent was obtained from participants and all protocols were approved by the institutional review boards of the participating institutions.

### Exposure

The primary exposure was eGFR, as estimated by the CKD‐EPI 2021 equation.^15^ The revised equation is as follows: eGFR_cr_ = 142 × min(S_cr_/κ, 1)^α^ × max(S_cr_/κ, 1)^−1.200^ × 0.9938^Age^ × 1.012 [if female], where S_cr_ = serum creatinine in mg/dL, κ = 0.7 (women) or 0.9 (men), α = −0.241 (women) or −0.302 (men), min(S_cr_/κ, 1) is the minimum of S_cr_/κ or 1.0, max(S_cr_/κ, 1) is the maximum of S_cr_/κ or 1.0, and age is in years.^16^ For categorical comparisons, kidney function was stratified into 4 levels based on cutoffs from the National Kidney Foundation: eGFR≥90 (normal, G1), eGFR 60 to 89 (mild, G2), eGFR 45 to 59 (mild–moderate, G3a), and eGFR <45 mL/min per 1.73 m^2^ (moderate–severe and severe, G3b–G5).

### Outcome

The primary outcome was incident HF, defined as the first hospitalization for acute decompensated HF, and prespecified for subgroups of HFrEF and HFpEF. All HF cases were adjudicated by WHI trained physician adjudicators using criteria for overall HF, HFrEF, and HFpEF, which have been described in detail elsewhere.^17^ Briefly, hospital records of suspected HF after self‐report of outcomes (semiannually during the clinical trials/observational studies phase between 1993 and 2010, annually from 2010 onward) were abstracted to include evidence of new onset of symptoms, history of HF, general medical history, physical examination signs and symptoms, diagnostic tests (chest radiograph, echocardiogram, cardiac catheterization, coronary angiography, cardiac radionuclide ventriculogram, cardiac magnetic resonance imaging, cardiac computed tomography scan, stress test), biomarkers (BNP [brain natriuretic peptide], NT‐proBNP [N‐terminal prohormone BNP], cardiac troponins), and medications. Physician adjudicators reviewed this information for evidence of acute decompensated HF. Adjudication of hospitalized HF subtypes (HFpEF, HFrEF, and HFmrEF [midrange EF]) was based on measured LVEF at the time of HF diagnosis. HFrEF was defined as HF with an EF <40% and HFpEF was defined as HF with an EF ≥50%, and HFmrEF between 40% and 49%. If no EF was available, it was classified as HF with unconfirmed EF. We previously have compared the subgroup of women with LVEF information to those without LVEF information and there were no substantial differences in major HF risk factors or other relevant characteristics.^18^ The acute HF classification system used in this analysis has been shown to have good agreement with other epidemiological HF algorithms.^19^

### Statistical Analysis

Baseline characteristics of the cohort are reported as means±SD, median and interquartile range, or frequencies and proportions for normally distributed continuous, nonnormally distributed continuous, and categorical variables, respectively. Wilcoxon rank sum tests, *t* tests, chi‐square tests for normally distributed continuous, nonnormally distributed continuous, and categorical variables, respectively, were used to compare characteristics between the different eGFR groups.

To determine linearity of association between eGFR and incident HF, age‐adjusted eGFR was plotted against log‐transformed hazard ratio for overall HF, HFrEF, and HFpEF with restricted cubic splines (knots were placed at 5%, 35%, 65%, and 95%) allowing 3 degrees of freedom.

Multivariable‐adjusted Cox proportional hazards models were used to assess the association of eGFR groups and incident HF. Hazard ratios (HRs) and 95% CIs were calculated from Cox models stratified by study membership (clinical trials arm or observational study arm). The proportional hazards assumption was evaluated using Schoenfeld residuals; there were no violations of this assumption. Multicollinearity between independent variables was assessed by calculating variance inflation factors. All variance inflation factors were below a cutoff of 5 suggesting no issue of multicollinearity. Follow‐up time was defined as days from WHI enrollment to incident HF for those with incident HF. For those without HF, follow‐up time was the number of days between enrollment and last follow‐up. Women were censored at death from any cause, loss to follow‐up, or the end of the follow‐up period (through 2021), whichever came first. Inverse probability weighting was used in all models to account for the sampling frame of the cohort using a logistic regression model to compute the probability of cohort inclusion incorporating age, body mass index, race, ethnicity, history of hysterectomy and length of follow‐up as covariates.

Baseline covariates were selected a priori based on clinical relevance and known association with HF from previous literature. Covariates in the adjusted models included model 1: age; model 2: age, race and ethnicity, marital status, income, education; model 3: model 2 covariables with addition of treated diabetes, treated hypertension, measured systolic and diastolic blood pressure, history of atrial fibrillation, history of hysterectomy, and history of coronary heart disease; model 4: model 3 with addition of body mass index, physical activity, smoking status, Healthy Eating index diet quality score, coffee intake, and alcohol intake.

Prespecified interaction terms for diabetes, hypertension, history of coronary heart disease, hormone therapy use (defined as self‐report of exposure to menopausal hormone replacement therapy with estrogen or progesterone or combination therapy at baseline), race, ethnicity, and age (<65 versus ≥65 years) were tested for the association of eGFR with incident HF.

We performed a complete case analysis. Inspection of the covariates for missingness showed almost all covariables had <5% missing, with the majority having <2% missing. As a final step in the analysis, we examined the difference in survival curves for HF subtypes (specifically, HFpEF and HFrEF). To test for this difference, the Lunn–McNeil method^20^ was used to test for heterogeneity of the HF subdistributions. An SAS macro developed by Wang et al. was used to calculate the likelihood ratio *P* value for heterogeneity.^21^ Income and education, which have historically had significant missing or unknown responses, were coded with a missing/unknown level. A 2‐sided *P* value of 0.05 was used to determine statistical significance. All analyses were performed using SAS Version 9.4 (SAS Institute, Cary, NC).

## RESULTS

The WHI included 161 808 participants. After exclusions for no available creatinine value, self‐reported HF at baseline, not part of the HF subcohort, or missing length of follow‐up (Figure 1), the final study cohort consisted of 23 309 participants. Creatinine was assessed in the biomarkers cohort of the WHI. When comparing individuals in the biomarkers compared with those not in the biomarkers cohort, the biomarkers cohort was oversampled intentionally for Black and Hispanic individuals. Other characteristics comparing the 2 cohorts are shown in Table S1.

### Characteristics by eGFR Group

The majority of the cohort had eGFR ≥90 (50.7%) or eGFR between 60 and 89 (43.7%). There were 4.5% of participants with eGFR between 45 and 59 and 1.1% had eGFR <45. Baseline characteristics are shown in Table 1. Participants with lower eGFR were slightly older and more likely to be age >70 years. For race and ethnicity, there was a higher proportion of non‐Hispanic White participants in the higher eGFR groups (eGFR 60–90 and eGFR ≥90). The lower eGFR group was less likely to be married or partnered, had a higher proportion with lower income, and had a higher proportion of less than high school education.

**Table 1:** Baseline Characteristics Stratified by eGFR

	Level 1 (eGFR<45)	Level 2 (45≤eGFR<60)	Level 3 (60≤eGFR<90)	Level 4 (eGFR≥90)	*P* trend
Mean serum creatinine±SD →	1.66 (0.58)	1.12 (0.08)	0.84 (0.08)	0.65 (0.07)	
	n=256	n=1048	n=10 191	n=11 814	
Demographics					
Age, y, mean±SD	68.2 (6.8)	67.6 (6.8)	65.5 (7.1)	62.3 (7.1)	<0.001
Age class, n (%)					
50–59 y	35 (13.7)	145 (13.8)	2274 (22.3)	4454 (37.7)	<0.001
60–69 y	103 (40.2)	441 (42.1)	4713 (46.3)	5389 (45.6)	0.224
≥70 y	118 (46.1)	462 (44.1)	3204 (31.4)	1971 (16.7)	<0.001
Race or ethnicity, n (%)					
Non‐Hispanic White	72 (28.4)	376 (36.0)	5061 (49.8)	5802 (49.2)	<0.001
Black	158 (62.2)	578 (55.3)	4004 (39.4)	3267 (27.7)	<0.001
Hispanic	22 (8.7)	78 (7.5)	940 (9.3)	2531 (21.5)	<0.001
Other^*^	2 (0.8)	13 (1.2)	155 (1.5)	189 (1.6)	0.345
Marital status (n, %)					<0.001
Married or partnered	100 (39.4)	442 (42.6)	5148 (50.8)	6617 (56.4)	
Socioeconomic status					
Income, n (%)					
$50 000 or greater	31 (12.1)	187 (17.8)	2510 (24.6)	3095 (26.2)	<0.001
$20 000 to <$50 000	96 (37.5)	437 (41.7)	4536 (44.5)	5279 (44.7)	0.042
Less than $20 000 per year	104 (40.6)	351 (33.5)	2494 (24.5)	2727 (23.1)	<0.001
Missing/don't know	25 (9.8)	73 (7.0)	651 (6.4)	713 (6.0)	0.024
Education, n (%)					
Less than high school graduate	52 (20.3)	156 (14.9)	887 (8.7)	1132 (9.6)	<0.001
High school graduate	33 (12.9)	176 (16.8)	1834 (18.0)	2159 (18.3)	0.066
Some college	101 (39.5)	392 (37.4)	3893 (38.2)	4765 (40.3)	0.002
College graduate	64 (25.0)	310 (29.6)	3498 (34.3)	3654 (30.9)	0.036
Missing	6 (2.3)	14 (1.3)	79 (0.8)	104 (0.9)	0.209
CHD risk factors					
Diabetes, n (%)	92 (36.1)	201 (19.2)	1174 (11.5)	1849 (15.7)	0.891
Hyperlipidemia, n (%)	69 (27.0)	237 (22.6)	1618 (15.9)	1538 (13.0)	<0.001
Hypertension, n (%)	208 (81.3)	661 (63.1)	4401 (43.2)	4222 (35.7)	<0.001
Cigarette smoking status, n (%)					
Never	122 (50.2)	570 (55.5)	5361 (53.4)	5973 (51.3)	0.007
Past	93 (38.3)	387 (37.7)	3874 (38.6)	4442 (38.2)	0.992
Current	28 (11.5)	70 (6.8)	813 (8.1)	1222 (10.5)	<0.001
Family history of CHD, n (%)	22 (8.6)	107 (10.2)	958 (9.4)	1167 (9.9)	0.393
BMI, kg/m^2^, mean±SD	31.4 (6.4)	30.5 (6.6)	29.6 (6.1)	29.6 (6.4)	<0.001
BMI categories, n (%)					
<25.0	34 (13.3)	191 (18.2)	2360 (23.2)	2871 (24.3)	<0.001
25 to <30	85 (33.2)	373 (35.6)	3700 (36.3)	4076 (34.5)	0.054
30 to <35	66 (25.8)	265 (25.3)	2478 (24.3)	2813 (23.8)	0.167
≥35	71 (27.7)	218 (20.8)	1650 (16.2)	2053 (17.4)	0.083
Obese (BMI ≥30 kg/m^2^), n (%)	137 (53.5)	483 (46.1)	4128 (40.5)	4866 (41.2)	0.011
Physiological measures, mean±SD					
Systolic blood pressure, mm Hg	139 (20.2)	135 (18.5)	131 (17.7)	129 (17.4)	<0.001
Diastolic blood pressure, mm Hg	76 (9.9)	76 (9.7)	76 (9.5)	76 (9.2)	0.437
Height, cm	160.5 (7.1)	161.5 (6.7)	161.6 (6.5)	160.7 (6.8)	<0.001
Lifestyle factors					
Physical activity, metabolic equivalent task‐h/wk, mean±SD	8.31 (11.12)	9.53 (12.31)	10.85 (13.03)	10.62 (13.17)	0.074
Alcohol, servings/wk, mean±SD	0.96 (3.04)	1.24 (3.98)	1.73 (4.37)	2.03 (5.35)	<0.001
Coffee drinker, n (%)	134 (53.6)	630 (60.5)	6905 (68.3)	8379 (71.5)	<0.001
Alternative healthy eating index 2010, mean±SD	47.3 (8.9)	48.3 (9.7)	49.7 (10.0)	49.7 (10.1)	<0.001
Calibrated total energy, calories/d, mean±SD	2240 (287.7)	2237 (278.3)	2267 (273.3)	2335 (285.2)	<0.001
Walking limitation—1 block, n (%)					
Yes, limited a lot	25 (10.0)	38 (3.7)	228 (2.3)	226 (1.9)	<0.001
Yes, limited a little	65 (25.9)	155 (15.1)	802 (8.0)	919 (7.9)	<0.001
No limitation	161 (64.1)	837 (81.3)	9032 (89.8)	10 517 (90.2)	<0.001
SF‐36 fatigue, mean±SD	54.7 (20.4)	60.4 (19.2)	63.9 (18.9)	63.0 (19.2)	0.002
SF‐36 physical functioning, mean±SD	59.4 (25.8)	71.3 (23.8)	78.5 (21.2)	79.7 (20.7)	<0.001
Care issues, n (%)					
Insurance	240 (95.6)	967 (94.2)	9482 (94.6)	10 509 (90.2)	<0.001
Regular health care provider	242 (95.3)	972 (94.5)	9213 (91.9)	10 452 (89.7)	<0.001
Medications, n (%)					
Aspirin	69 (27.0)	204 (19.5)	2044 (20.1)	2133 (18.1)	<0.001
Diuretics	130 (50.8)	379 (36.2)	1959 (19.2)	1523 (12.9)	<0.001
Antihypertensive meds	94 (36.7)	246 (23.5)	1385 (13.6)	1230 (10.4)	<0.001
Angiotensin‐converting enzyme inhibitor	67 (26.2)	164 (15.7)	1004 (9.9)	891 (7.5)	<0.001
Angiotensin receptor blocker	10 (3.9)	20 (1.9)	61 (0.6)	58 (0.5)	<0.001
Beta blocker	32 (12.5)	147 (14.0)	870 (8.5)	784 (6.6)	<0.001
Calcium channel blocker	87 (34.0)	208 (19.9)	1365 (13.4)	1330 (11.3)	<0.001
Lipid‐lowering meds	48 (18.8)	139 (13.3)	959 (9.4)	855 (7.2)	<0.001
Multivitamin	63 (24.6)	319 (30.4)	3293 (32.3)	3857 (32.7)	0.030
Hormone treatment					
Never	145 (57.5)	549 (53.7)	4903 (49.2)	5323 (46.3)	<0.001
Past	66 (26.2)	304 (29.7)	3295 (33.0)	3541 (30.8)	0.146
Current	41 (16.3)	170 (16.6)	1778 (17.8)	2628 (22.9)	<0.001
Medical history at baseline, n (%)					
CHD	27 (10.6)	67 (6.4)	364 (3.6)	313 (2.7)	<0.001
Chronic obstructive pulmonary disease	15 (6.2)	61 (6.1)	365 (3.8)	392 (3.6)	0.001
Atrial fibrillation	17 (6.8)	65 (6.3)	435 (4.4)	393 (3.4)	<0.001
Anemia, hemoglobin < 11 gm/dL	30 (11.9)	30 (2.9)	111 (1.1)	96 (0.8)	<0.001
Hysterectomy	136 (53.1)	515 (49.1)	4520 (44.4)	5423 (45.9)	0.618
Dialysis history during follow‐up	12 (4.7)	16 (1.5)	35 (0.3)	23 (0.2)	<0.001
Number of comorbid conditions					
0	66 (25.8)	502 (47.9)	6129 (60.1)	7276 (61.6)	<0.001
1	87 (34.0)	279 (26.6)	2540 (24.9)	2855 (24.2)	0.002
2+	103 (40.2)	267 (25.5)	1522 (14.9)	1683 (14.3)	<0.001
Outcomes, n (%)					
Any HF	58 (22.7)	142 (13.6)	1019 (10.0)	993 (8.4)	<0.001
Preserved ejection fraction HF	30 (11.7)	67 (6.4)	519 (5.1)	491 (4.2)	<0.001
Reduced ejection fraction HF	10 (3.9)	39 (3.7)	229 (2.3)	233 (2.0)	0.002

BMI indicates body mass index; CHD, coronary heart disease; eGFR, estimated glomerular filtration rate; HF, heart failure; and SF‐36, Short Form‐36.

^*^ Other indicates Native American or Alaska Native, Asian, and Native Hawaiian or Other Pacific Islander.

For cardiovascular risk factors (Table 1), those with lower eGFR had higher prevalence of diabetes, hyperlipidemia, and hypertension, with a higher proportion with obesity (defined by body mass index ≥30 kg/m^2^) compared with higher eGFR. The lower eGFR group had a higher proportion of other medical conditions, including a higher proportion with 2+ comorbidities.

There was no significant difference in physical activity or alcohol use for those with high compared with low eGFR, although those with lower eGFR had lower RAND‐36 Item Health Survey physical functioning estimates (Table 1). Those in the lowest and highest eGFR groups were more likely to be current cigarette smokers. Those with higher eGFR were less likely to be insured. Those with lower eGFR were more likely to be on aspirin, diuretics, antihypertensives, and lipid‐lowering drugs. There was a higher proportion of never hormone therapy use in those with lower eGFR.

### Age‐Adjusted Incidence HF Rates

Median follow‐up time for the total cohort was 18.8 years (interquartile range 11.3 years). During follow‐up, there were a total of 8714 deaths over 393 863 person‐years giving an age‐adjusted death rate of 23.4 (per 1000 person‐years).

**Figure 2:**
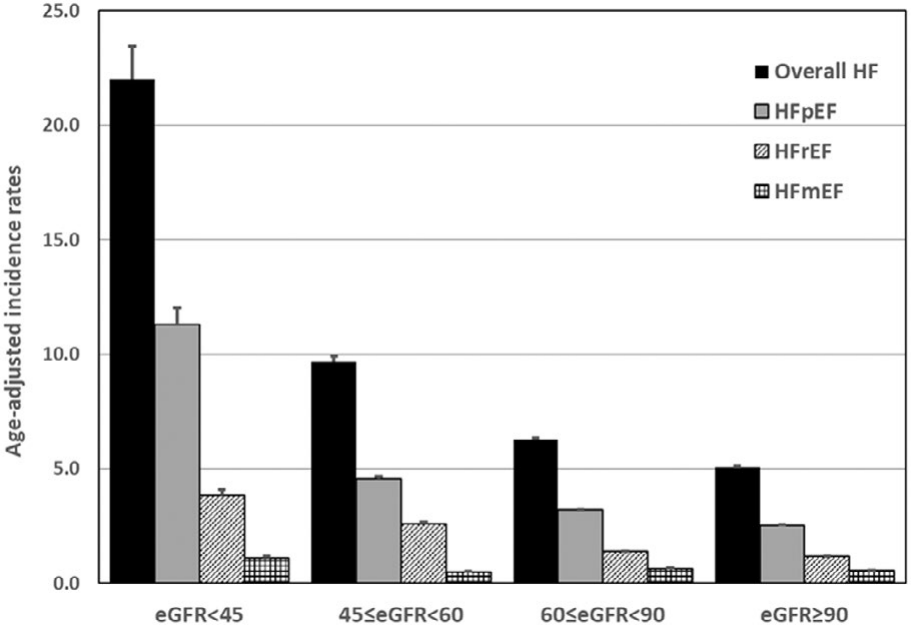
Age‐adjusted incidence rates per 1000 person‐years for heart failure and its subtypes by eGFR level. Incidence rates for each type of heart failure increased with declining eGFR groups. eGFR indicates estimated glomerular filtration rate; HF, heart failure; HFmEF, heart failure with mid‐range ejection fraction; HFpEF, heart failure with preserved ejection fraction; and HFrEF, heart failure with reduced ejection fraction.

Of the 23 309 participants evaluated, there were 2212 cases of incident HF, of which 1107 were HFpEF (50.0%), 511 were HFrEF (23.1%), 226 cases were HFmrEF (10.2%), and 368 cases had undetermined LVEF (16.6%) (Table S2). Crude, age‐specific, and age‐adjusted incidence HF rates are shown in Table 2. Age‐adjusted incidence rates (per 1000 person‐years) of overall HF were 5.1 for eGFR >90, 6.3 for eGFR 60 to 89, 9.7 for eGFR 45 to 60, and 22.0 for eGFR <45. Age‐adjusted incidence rates for HF stratified into HFrEF, HFpEF, and HFmrEF are depicted in Figure 2.

**Table 2:** Age‐Adjusted Incidence Rates of Heart Failure Stratified by eGFR

Outcomes	eGFR<45	45≤eGFR<60	60≤eGFR<90	eGFR≥90
Any HF				
Crude HF incidence rate, #/1000 person‐years	21.52 (20.72– 22.35)	9.34 (9.20– 9.49)	5.96 (5.93– 5.99)	4.84 (4.82– 4.86)
Age‐specific HF incidence rates, #/1000 person‐years				
50–59	20.31 (18.50– 22.29)	4.87 (4.69– 5.06)	2.60 (2.58– 2.62)	2.30 (2.29– 2.32)
60–69	16.44 (15.52– 17.42)	8.87 (8.66– 9.09)	5.32 (5.28– 5.36)	5.59 (5.55– 5.62)
70+	27.36 (25.79– 29.03)	11.91 (11.61– 12.21)	10.30 (10.21– 10.40)	10.00 (9.88– 10.11)
Age‐adjusted HF rates	22.00 (20.66– 23.44)	9.66 (9.41– 9.91)	6.28 (6.23– 6.33)	5.08 (5.04– 5.12)
HFpEF				
Crude HFpEF incidence rate, #/1000 person‐years	11.13 (10.72– 11.56)	4.41 (4.34– 4.48)	3.05 (3.04– 3.07)	2.41 (2.40– 2.42)
Age‐specific HFpEF incidence rates, #/1000 person‐years				
50–59	13.54 (12.34– 14.86)	1.87 (1.80– 1.95)	1.48 (1.47– 1.50)	1.06 (1.05– 1.06)
60–69	7.79 (7.35– 8.25)	4.36 (4.26– 4.47)	2.71 (2.69– 2.73)	2.83 (2.81– 2.84)
70+	13.68 (12.89– 14.51)	5.61 (5.47– 5.76)	5.16 (5.11– 5.21)	5.09 (5.03– 5.15)
Age‐adjusted HFpEF rates	11.29 (10.59– 12.04)	4.57 (4.45– 4.69)	3.21 (3.18– 3.23)	2.54 (2.51– 2.56)
HFrEF				
Crude HFrEF incidence rate, #/1000 person‐years	3.71 (3.57– 3.85)	2.57 (2.53– 2.61)	1.34 (1.33– 1.35)	1.14 (1.13– 1.14)
Age‐specific HFrEF incidence rates, #/1000 person‐years				
50–59	4.51 (4.11– 4.95)	1.87 (1.80– 1.95)	0.64 (0.63– 0.64)	0.72 (0.72– 0.73)
60–69	1.73 (1.63– 1.83)	2.56 (2.50– 2.62)	1.29 (1.28– 1.30)	1.26 (1.25– 1.26)
70+	5.47 (5.16– 5.81)	2.89 (2.82– 2.97)	2.09 (2.07– 2.11)	1.98 (1.96– 2.00)
Age‐adjusted HFrEF rates	3.84 (3.60– 4.09)	2.61 (2.54– 2.68)	1.40 (1.39– 1.41)	1.18 (1.17– 1.18)
HFmEF				
Crude HFmEF incidence rate, #/1000 person‐years	1.11 (1.07– 1.16)	0.46 (0.45– 0.47)	0.61 (0.61– 0.62)	0.54 (0.54– 0.54)
Age‐specific HFmEF incidence rates, #/1000 person‐years				
50–59	0.00 (0.00– 0.00)	0.00 (0.00– 0.00)	0.21 (0.20– 0.21)	0.18 (0.18– 0.18)
60–69	1.73 (1.63– 1.83)	0.30 (0.29– 0.31)	0.57 (0.56– 0.57)	0.71 (0.71– 0.72)
70+	0.91 (0.86– 0.97)	0.85 (0.83– 0.87)	1.09 (1.08– 1.10)	1.06 (1.05– 1.07)
Age‐adjusted HFmEF rates	1.12 (1.05– 1.18)	0.50 (0.49– 0.51)	0.65 (0.64– 0.66)	0.57 (0.57– 0.57)

eGFR indicates estimated glomerular filtration rate; HF, heart failure; HFmEF, heart failure with midrange ejection fraction; HFpEF, heart failure with preserved ejection fraction; and HFrEF, heart failure with reduced ejection fraction.

### Spline Analysis

**Figure 3:**
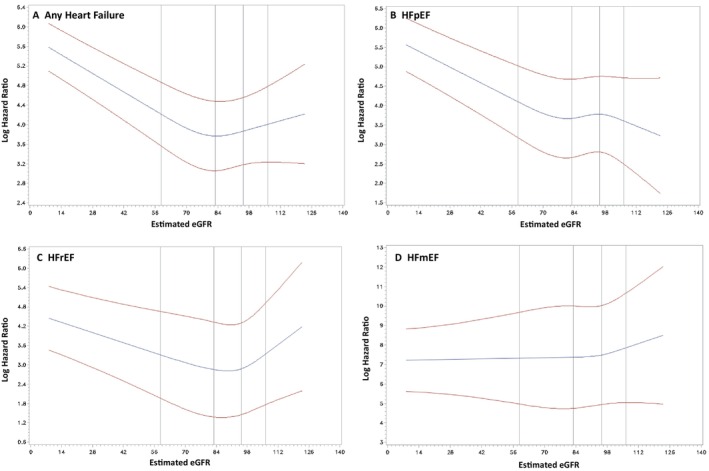
Spline analysis for eGFR risk and heart failure (adjusted for age). The *y* axis is log hazard ratio and *x* axis is eGFR. Estimated splines (blue lines) with their 95% CIs (red lines) are shown for (**A**) Any incident heart failure (*P*<0.001 for linearity); (**B**) Heart failure with preserved ejection fraction (*P*<0.001 for linearity); (**C**) Heart failure with reduced ejection fraction (*P*<0.001 for linearity); (**D**) Heart failure with midrange ejection fraction (*P*, 0.484 for linearity). eGFR indicates estimated glomerular filtration rate; HFmEF, heart failure with mid‐range ejection fraction; HFpEF, heart failure with preserved ejection fraction; and HFrEF, heart failure with reduced ejection fraction.

Age‐adjusted restricted cubic splines showed a linear association between eGFR and overall HF until eGFR is approximately 84 (Figure 3A). The relationship with incident HFpEF is mostly linear through the full range of eGFR values (Figure 3B), whereas the relationship with HFrEF is linear up to an eGFR of 84 (Figure 3C). There appeared to be a potential U‐shaped relationship of HFrEF with kidney function for eGFR >98 with higher eGFR associated with increasing risk for incident HFrEF, although this was seen in the more extreme values likely driven by instability of the estimates of the splines at the tails because of lower n at the extreme values. Although the relationship was linear for eGFR and incident HFmrEF, there was no apparent association between eGFR and incident HFmrEF (Figure 3D), but these results should be interpreted with caution due to the limited sample size relative to the other HF subgroups.

### Association of CKD With Incident HF

For each 10‐unit decrease in eGFR, risk for overall incident HF was 7% higher in the fully adjusted model (HR, 1.07 [95% CI, 1.06–1.09]) (Table 3). When analyzed as a categorical eGFR exposure variable, there was no significant difference in HF risk between eGFR ≥90 and eGFR 60 to 89 in either the unadjusted or fully adjusted models. However, when compared with eGFR ≥90, there was a significant association of eGFR 45 to 59 with incident HF (fully adjusted HR, 1.36 [95% CI, 1.26–1.47]) and further increased risk for eGFR <45 (fully adjusted HR, 2.46 [95% CI, 2.18–2.77]).

**Table 3:** Cox Proportional Hazard Models for Association of eGFR With Heart Failure

	HR (95% CI)	eGFR range	eGFR<45	eGFR 45–59	eGFR 60–89	eGFR ≥90	*P* trend
Model	Per 10 eGFR unit decrease	eGFR median	38.51 HR (95% CI)	54.85 HR (95% CI)	78.72 HR (95% CI)	98.15	
Any heart failure							
Age adjusted	1.08 (1.06–1.09)		4.28 (3.84–4.77)	1.57 (1.46–1.69)	0.92 (0.89–0.95)	(ref)	<0.001
Fully adjusted*	1.07 (1.06–1.09)		2.46 (2.18–2.78)	1.36 (1.26–1.47)	1.02 (0.99–1.06)	(ref)	<0.001
HFpEF							
Age adjusted	1.13 (1.11–1.15)		4.94 (4.21–5.79)	1.76 (1.59–1.95)	0.99 (0.94–1.04)	(ref)	<0.001
Fully adjusted*	1.12 (1.10–1.14)		2.80 (2.36–3.32)	1.51 (1.36–1.69)	1.07 (1.01–1.12)	(ref)	<0.001
HFrEF							
Age adjusted	1.02 (1.00–1.05)		3.21 (2.47–4.17)	1.70 (1.47–1.97)	0.91 (0.85–0.97)	(ref)	<0.001
Fully adjusted*	1.03 (1.00–1.05)		2.18 (1.66–2.87)	1.51 (1.29–1.76)	1.06 (0.99–1.14)	(ref)	<0.001
HFmEF							
Age adjusted	0.88 (0.84–0.91)		0.61 (0.27–1.37)	0.79 (0.60–1.04)	0.76 (0.69–0.84)	(ref)	<0.001
Fully adjusted*	0.90 (0.86–0.93)		0.14 (0.03–0.60)	0.74 (0.56–0.98)	0.89 (0.81–0.99)	(ref)	<0.001

* Adjusted for age, race, ethnicity, marital status, income, education, diabetesellitus, hypertension, systolic blood pressure, diastolic blood pressure, atrial fibrillation, hysterectomy, history of coronary heart disease, body mass index, physical activity, smoking status, diet quality, coffee intake, alcohol intake, and ability to walk 1 block. eGFR indicates estimated glomerular filtration rate; HFmEF, heart failure with midrange ejection fraction; HFpEF, heart failure with preserved ejection fraction; HFrEF, heart failure with reduced ejection fraction; and HR, hazard ratio.

Evaluating HF subtypes (Table 3), similar findings were seen in HFpEF compared with total HF, with a stepwise increase in risk by CKD stage once eGFR was 45 to 59 or lower. Similar findings were again seen for the group with HFrEF. A comparison of HFpEF and HFrEF subdistributions showed a significant overall difference in the association of eGFR level and HF subtype (*P*=0.017). This difference was most apparent in eGFR <45 level (*P*=0.014). Findings for HFmrEF were discordant with the other groups but with unstable estimates due to low event rates in the lower eGFR groups (7 incident HFmrEF events for eGFR 45–60 and 3 events for eGFR <45).

### Exploratory Subgroup Analyses

**Figure 4:**
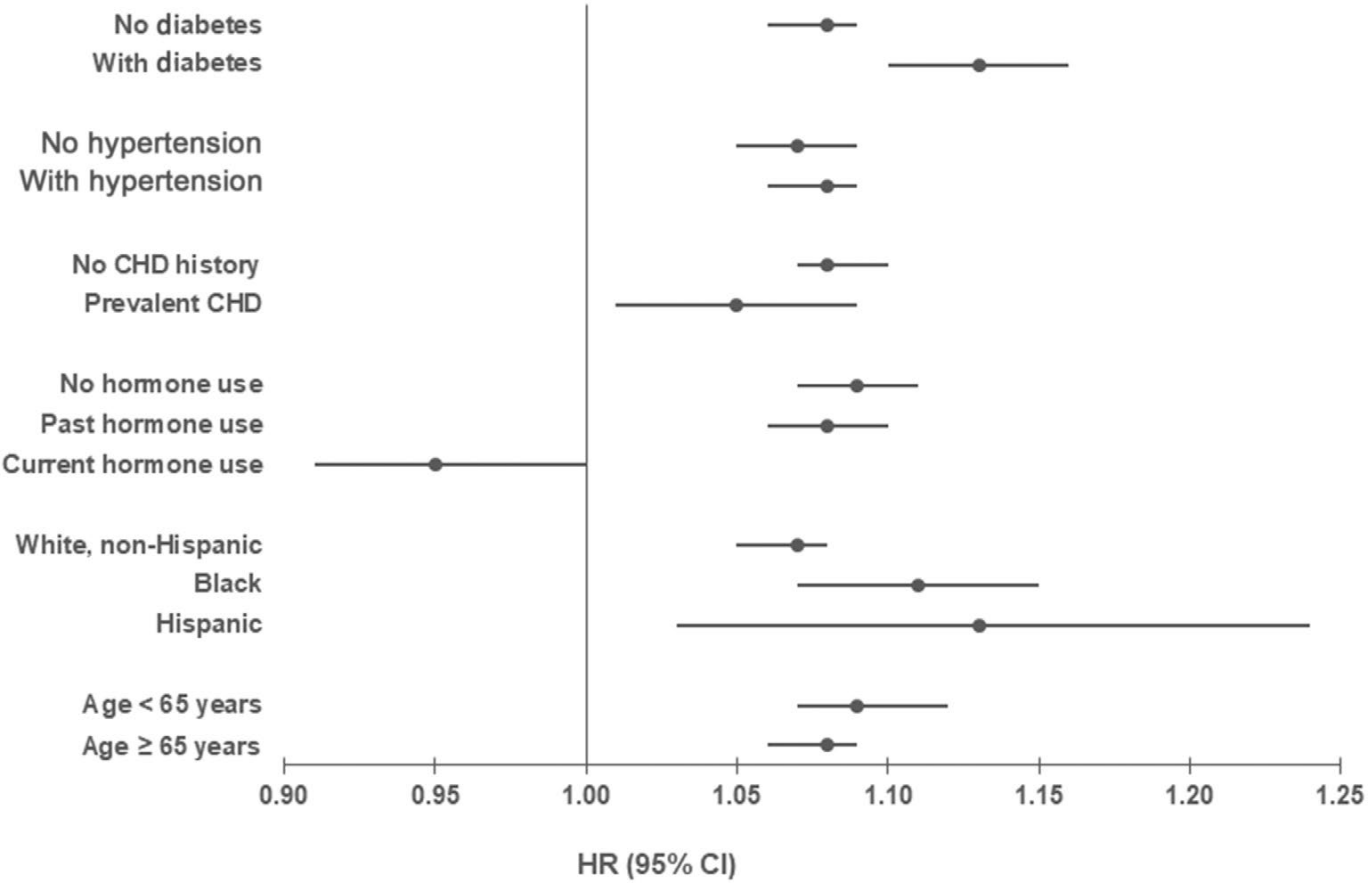
Forest plot of fully adjusted* hazard ratios for each 10‐unit decrease in eGFR (95% CIs) for overall heart failure stratified by selected risk factors. *Adjusted for age, race, ethnicity, marital status, income, education, diabetes, hypertension, systolic blood pressure, diastolic blood pressure, atrial fibrillation, hysterectomy, history of coronary heart disease, body mass index, physical activity, smoking status, diet quality, coffee intake, alcohol intake, and ability to walk 1 block (note—risk factor excluded from model when examined). CHD indicates coronary heart disease; eGFR, estimated glomerular filtration rate; and HR, hazard ratio.

Findings were consistent across prespecified subgroup analyses for a history of hypertension, prevalent coronary heart disease, race and ethnicity, and age threshold of 65 years (Figure 4 and Table S3). However, the association of CKD with incident HF was stronger in those with a history of diabetes (interaction *P*<0.01). For those with a history of current hormone use, the association of CKD with incident HF appeared to be attenuated compared with those with no hormone use or past hormone use (interaction *P*=0.01).

## DISCUSSION

In the current study that prospectively examined in postmenopausal women the risk of incident HF associated with eGFR as a measure of kidney function, we found the following: (1) Restricted cubic splines suggested lower eGFR was associated with increased risk of overall incident HF. The association of eGFR with incident HFpEF appeared to be mostly linear through the full range of eGFR, whereas for HFrEF the association was linear at lower eGFR but less clear at higher eGFR values. (2) There was stepwise increased risk with progressively lower eGFR categories for incident HF, HFpEF, and HFrEF. However, the association of eGFR with incident HF was stronger for HFpEF than for HFrEF. (3) For prespecified subgroup analyses, the association of eGFR with incident HF was stronger in those with diabetes compared with those without diabetes. Interestingly, the use of current hormone use appeared to attenuate the association compared with those without hormone use or prior hormone use.

Prior studies have demonstrated the association of CKD with incident HF with threshold eGFR <45^10^ or eGFR <60.^11^, ^12^ A recent study from the CKD Prognosis Consortium assessed the association of eGFR and albuminuria with adverse kidney and cardiovascular outcomes in over 27 million individuals from 114 global cohorts. There was a stepwise increase in risk for incident HF for categories of eGFR <90, as well as increasing risk with higher albumin‐creatinine ratio.^22^ Our results are consistent with these other studies in demonstrating an association of declining eGFR with increased risk for incident HF. Due to the phenotyping of HF subtypes available in the WHI, we were able to individually assess the association of kidney function with HFrEF and HFpEF. Unlike the threshold effect seen in HFrEF, there was a consistent association of declining eGFR with increased risk for HFpEF throughout the full range of eGFR. Additionally, although there was an association of eGFR with both HFrEF and HFpEF, the point estimate for the risk for HFpEF with eGFR <45 was stronger and provides additional evidence for derangement of the cardiorenal axis as a contributor to incident HFpEF. Interestingly, we did not find a significant association of eGFR with HFmrEF, though this may be related to the relatively smaller sample size of this group compared with the HFrEF and HFpEF groups rather than a true lack of association.

The higher risk of CKD with incident HFpEF is consistent with putative mechanisms of myocardial pathogenesis in the setting of kidney dysfunction. In the case of HFpEF, a combination of interstitial fibrosis, cardiomyocyte hypertrophy and impaired calcium homeostasis contribute to the phenotype.^23^, ^24^ With kidney dysfunction, there are multiple mechanisms that may lead to these changes. First, there are shared comorbidities of hypertension, diabetes, and obesity common to cardiovascular disease and CKD; this underscores the detrimental cardiovascular effects of cardiometabolic abnormalities. Second, vascular changes including calcification and increased arterial stiffening occur with kidney dysfunction. Third, systemic inflammation is driven by accumulation of glycation end products and uremic toxins, metabolic acidosis, insulin resistance, and proteinuria. Lastly, neurohormonal abnormalities in the renin‐angiotensin‐aldosterone pathway and sympathetic nervous system seen in HF also occur with CKD including impairment of sodium homeostasis.^1^, ^2^, ^3^ The strong association of CKD with HFpEF leads to the question of whether recognition of high‐risk individuals early in the CKD process may guide more intensive monitoring or focused intervention in these specific subgroups to abrogate downstream adverse outcomes. In particular, sodium‐glucose cotransporter 2 inhibitors mitigate the decline in eGFR for individuals with CKD^25^, ^26^ and dapagliflozin reduces the risk for HF hospitalization or death from cardiovascular causes in those with CKD.^26^

The association of CKD with incident HF appears to be stronger in those with diabetes as compared with those without diabetes. It is well established that diabetes substantially increases the risk of incident HF, believed to be due to dysregulation of many cellular mechanism including increased oxidative stress, inflammation, aberrant insulin signaling, accumulation of advanced glycated end‐products, alterations in myocardial substrate metabolism and altered signal transduction.^27^ Similarly, CKD is associated with inflammation^28^ and can result in increased glycated end‐products,^29^ potentiating the effects of CKD on the myocardium.

Interestingly, current menopausal hormone use appeared to attenuate the association of CKD with incident HF. In prior studies from the Women's Health Initiative, randomization to menopausal hormone therapy was not associated with HF risk.^30^ However, the association of hormone therapy with cardiovascular disease is complex and may vary depending on timing of use and with an age‐dependent effect modification.^31^ Inference regarding the benefits or risks of hormone replacement therapy should be based on randomized clinical trials and caution should be applied when interpreting retrospective self‐reported hormone therapy analyses, which may be prone to recall bias, selection and confounding bias.

## STUDY LIMITATIONS

We recognize that our study has several limitations. As with any observational study, there is risk for residual or unmeasured confounding. Misclassification of exposure or outcome is possible, but less likely in a study where end points were ascertained as part of a clinical trial and a long‐term observational cohort. We assessed baseline eGFR rather than sequential measurements for the association with HF. We did not have serum cystatin C or urine albumin‐to‐creatinine measurements available and kidney function was estimated from serum creatinine. Additionally, not all participants in the WHI had LVEF data available, so the current study involves a subset of the original WHI cohort; despite this, the study cohort is large and included more than 23 000 participants.

## CONCLUSIONS

Kidney dysfunction was associated with incident HF in postmenopausal women, with progressively higher risk for HF with lower eGFR categories. Although declining eGFR was associated with both HFrEF and HFpEF, the association was stronger with HFpEF. Future studies are needed to confirm these findings and assess whether targeting mechanisms of HF in individuals with kidney dysfunction may be beneficial.

## Sources of Funding

The WHI program is funded by the National Heart, Lung, and Blood Institute, National Institutes of Health, US Department of Health and Human Services through 75N92021D00001, 75N92021D00002, 75N92021D00003, 75N92021D00004, 75N92021D00005. Publication of this study was partially funded by an Alpha Phi grant.

## Disclosures

Taufiq Salahuddin is supported by National Heart, Lung, and Blood Institute grant 5T32HL007828‐24. The remaining authors have no disclosures to report.

## Supporting information

Tables S1–S3

## References

[1] Jankowski J , Floege J , Fliser D , Bohm M , Marx N . Cardiovascular disease in chronic kidney disease: pathophysiological insights and therapeutic options. Circulation. 2021;143:1157–1172. doi: 10.1161/CIRCULATIONAHA.120.050686 33720773 PMC7969169

[2] van de Wouw J , Broekhuizen M , Sorop O , Joles JA , Verhaar MC , Duncker DJ , Danser AHJ , Merkus D . Chronic kidney disease as a risk factor for heart failure with preserved ejection fraction: a focus on microcirculatory factors and therapeutic targets. Front Physiol. 2019;10:1108. doi: 10.3389/fphys.2019.01108 31551803 PMC6737277

[3] Paulus WJ , Tschope C . A novel paradigm for heart failure with preserved ejection fraction: comorbidities drive myocardial dysfunction and remodeling through coronary microvascular endothelial inflammation. J Am Coll Cardiol. 2013;62:263–271. doi: 10.1016/j.jacc.2013.02.092 23684677

[4] Sidney S , Go AS , Jaffe MG , Solomon MD , Ambrosy AP , Rana JS . Association between aging of the US population and heart disease mortality from 2011 to 2017. JAMA Cardiol. 2019;4:1280–1286. doi: 10.1001/jamacardio.2019.4187 31663094 PMC6822092

[5] Carrero JJ , Hecking M , Chesnaye NC , Jager KJ . Sex and gender disparities in the epidemiology and outcomes of chronic kidney disease. Nat Rev Nephrol. 2018;14:151–164. doi: 10.1038/nrneph.2017.181 29355169

[6] Lam CSP , Arnott C , Beale AL , Chandramouli C , Hilfiker‐Kleiner D , Kaye DM , Ky B , Santema BT , Sliwa K , Voors AA . Sex differences in heart failure. Eur Heart J. 2019;40:3859–3868c. doi: 10.1093/eurheartj/ehz835 31800034

[7] Vinson AJ , Collister D , Ahmed S , Tennankore K . Underrepresentation of women in recent landmark kidney trials: the gender gap prevails. Kidney Int Rep. 2022;7:2526–2529. doi: 10.1016/j.ekir.2022.08.022 36531883 PMC9751686

[8] Jin X , Chandramouli C , Allocco B , Gong E , Lam CSP , Yan LL . Women's participation in cardiovascular clinical trials from 2010 to 2017. Circulation. 2020;141:540–548. doi: 10.1161/CIRCULATIONAHA.119.043594 32065763

[9] Ravani P , Quinn R , Fiocco M , Liu P , Al‐Wahsh H , Lam N , Hemmelgarn BR , Manns BJ , James MT , Joanette Y , et al. Association of age with risk of kidney failure in adults with stage IV chronic kidney disease in Canada. JAMA Netw Open. 2020;3:e2017150. doi: 10.1001/jamanetworkopen.2020.17150 32945876 PMC7501537

[10] Bowling CB , Feller MA , Mujib M , Pawar PP , Zhang Y , Ekundayo OJ , Aban IB , Love TE , Sanders PW , Anker SD , et al. Relationship between stage of kidney disease and incident heart failure in older adults. Am J Nephrol. 2011;34:135–141. doi: 10.1159/000328905 21734366 PMC3136373

[11] Kottgen A , Russell SD , Loehr LR , Crainiceanu CM , Rosamond WD , Chang PP , Chambless LE , Coresh J . Reduced kidney function as a risk factor for incident heart failure: the Atherosclerosis Risk in Communities (ARIC) study. J Am Soc Nephrol. 2007;18:1307–1315. doi: 10.1681/ASN.2006101159 17344421

[12] Bansal N , Katz R , Robinson‐Cohen C , Odden MC , Dalrymple L , Shlipak MG , Sarnak MJ , Siscovick DS , Zelnick L , Psaty BM , et al. Absolute rates of heart failure, coronary heart disease, and stroke in chronic kidney disease: an analysis of 3 community‐based cohort studies. JAMA Cardiol. 2017;2:314–318. doi: 10.1001/jamacardio.2016.4652 28002548 PMC5832350

[13] Design of the Women's Health Initiative Clinical Trial and Observational Study . The Women's Health Initiative Study Group. Control Clin Trials. 1998;19:61–109. doi: 10.1016/S0197-2456(97)00078-0 9492970

[14] Hays J , Hunt JR , Hubbell FA , Anderson GL , Limacher M , Allen C , Rossouw JE . The Women's Health Initiative recruitment methods and results. Ann Epidemiol. 2003;13:S18–S77. doi: 10.1016/S1047-2797(03)00042-5 14575939

[15] Inker LA , Eneanya ND , Coresh J , Tighiouart H , Wang D , Sang Y , Crews DC , Doria A , Estrella MM , Froissart M , et al. New creatinine‐ and cystatin C‐based equations to estimate GFR without race. N Engl J Med. 2021;385:1737–1749. doi: 10.1056/NEJMoa2102953 34554658 PMC8822996

[16] National Kidney Foundation . CKD‐EPI Creatinine Equation (2021) . Accessed April 24. https://www.kidney.org/content/ckd‐epi‐creatinine‐equation‐2021.

[17] Eaton CB , Pettinger M , Rossouw J , Martin LW , Foraker R , Quddus A , Liu S , Wampler NS , Hank Wu WC , Manson JE , et al. Risk factors for incident hospitalized heart failure with preserved versus reduced ejection fraction in a multiracial cohort of postmenopausal women. Circ Heart Fail. 2016;9:e002883. doi: 10.1161/CIRCHEARTFAILURE.115.002883 27682440 PMC5111360

[18] Pandey A , LaMonte M , Klein L , Ayers C , Psaty BM , Eaton CB , Allen NB , de Lemos JA , Carnethon M , Greenland P , et al. Relationship between physical activity, body mass index, and risk of heart failure. J Am Coll Cardiol. 2017;69:1129–1142. doi: 10.1016/j.jacc.2016.11.081 28254175 PMC5848099

[19] Rosamond WD , Chang PP , Baggett C , Johnson A , Bertoni AG , Shahar E , Deswal A , Heiss G , Chambless LE . Classification of heart failure in the Atherosclerosis Risk in Communities (ARIC) study: a comparison of diagnostic criteria. Circ Heart Fail. 2012;5:152–159. doi: 10.1161/CIRCHEARTFAILURE.111.963199 22271752 PMC3326579

[20] Lunn M , McNeil D . Applying Cox regression to competing risks. Biometrics. 1995;51:524–532. doi: 10.2307/2532940 7662841

[21] Wang M , Spiegelman D , Kuchiba A , Lochhead P , Kim S , Chan AT , Poole EM , Tamimi R , Tworoger SS , Giovannucci E , et al. Statistical methods for studying disease subtype heterogeneity. Stat Med. 2016;35:782–800. doi: 10.1002/sim.6793 26619806 PMC4728021

[22] Writing Group for the CKDPC , Grams ME , Coresh J , Matsushita K , Ballew SH , Sang Y , Surapaneni A , Alencar de Pinho N , Anderson A , Appel LJ , et al. Estimated glomerular filtration rate, albuminuria, and adverse outcomes: an individual‐participant data meta‐analysis. JAMA. 2023;330:1266–1277. doi: 10.1001/jama.2023.17002 37787795 PMC10548311

[23] Frisk M , Le C , Shen X , Roe AT , Hou Y , Manfra O , Silva GJJ , van Hout I , Norden ES , Aronsen JM , et al. Etiology‐dependent impairment of diastolic cardiomyocyte calcium homeostasis in heart failure with preserved ejection fraction. J Am Coll Cardiol. 2021;77:405–419. doi: 10.1016/j.jacc.2020.11.044 33509397 PMC7840890

[24] Borlaug BA . The pathophysiology of heart failure with preserved ejection fraction. Nat Rev Cardiol. 2014;11:507–515. doi: 10.1038/nrcardio.2014.83 24958077

[25] The E‐KCG , Herrington WG , Staplin N , Wanner C , Green JB , Hauske SJ , Emberson JR , Preiss D , Judge P , Mayne KJ , et al. Empagliflozin in patients with chronic kidney disease. N Engl J Med. 2023;388:117–127. doi: 10.1056/NEJMoa2204233 36331190 PMC7614055

[26] Heerspink HJL , Stefansson BV , Correa‐Rotter R , Chertow GM , Greene T , Hou FF , Mann JFE , McMurray JJV , Lindberg M , Rossing P , et al. Dapagliflozin in patients with chronic kidney disease. N Engl J Med. 2020;383:1436–1446. doi: 10.1056/NEJMoa2024816 32970396

[27] Kenny HC , Abel ED . Heart failure in type 2 diabetes mellitus. Circ Res. 2019;124:121–141. doi: 10.1161/CIRCRESAHA.118.311371 30605420 PMC6447311

[28] Zoccali C , Vanholder R , Massy ZA , Ortiz A , Sarafidis P , Dekker FW , Fliser D , Fouque D , Heine GH , Jager KJ , et al. The systemic nature of CKD. Nat Rev Nephrol. 2017;13:344–358. doi: 10.1038/nrneph.2017.52 28435157

[29] Stinghen AE , Massy ZA , Vlassara H , Striker GE , Boullier A . Uremic toxicity of advanced glycation end products in CKD. J Am Soc Nephrol. 2016;27:354–370. doi: 10.1681/ASN.2014101047 26311460 PMC4731113

[30] Liu L , Klein L , Eaton C , Panjrath G , Martin LW , Chae CU , Greenland P , Lloyd‐Jones DM , Wactawski‐Wende J , Manson JE . Menopausal hormone therapy and risks of first hospitalized heart failure and its subtypes during the intervention and extended postintervention follow‐up of the women's health initiative randomized trials. J Card Fail. 2020;26:2–12. doi: 10.1016/j.cardfail.2019.09.006 31536806

[31] Chester RC , Kling JM , Manson JE . What the Women's Health Initiative has taught us about menopausal hormone therapy. Clin Cardiol. 2018;41:247–252. doi: 10.1002/clc.22891 29493798 PMC6490107

