# Supplementary material for: Association of Kidney Function With Incident Heart Failure: An Analysis of the Women's Health Initiative
Source: J Am Heart Assoc. 2025 Feb 25;14(5):e037051. doi: 10.1161/JAHA.124.037051 (PMC12132762; doi:10.1161/JAHA.124.037051)
Supplement: Supplementary file 1 — Tables S1–S3 [file JAH3-14-e037051-s001.pdf]

SUPPLEMENTAL MATERIAL

Table S1. Comparison of baseline characteristics of women in the CVD biomarkers cohort to those not in the biomarkers cohort.

	Member of CVD Biomarkers Cohort		
	No (n=137,603)	Yes (n=24,205)	*p-value*
Demographics			
Age *(years) (mean, sd)*	63.1 (7.2)	64.0 (7.3)	<.0001
Age class *(n, %)*			<.0001
50-59 years	46364 (33.7)	7195 (29.7)	
60-69 years	61554 (44.7)	11035 (45.6)	
70+ years	29685 (21.6)	5975 (24.7)	
Race *(n, %)*			<.0001
Caucasian	121789 (89.0)	11539 (47.8)	
African American	5909 (4.3)	8258 (34.2)	
Hispanic	3681 (2.7)	3631 (15.0)	
Asian	3961 (2.9)	4 (0.0)	
Other	1567 (1.1)	708 (2.9)	
Marital status *(n, %)*			
Married or partnered	87527 (63.9)	12731 (52.9)	<.0001
SES			
Income *(n, %)*			<.0001
$50,000 or greater	51939 (37.8)	5982 (24.7)	
$20,000 - <$50,000	56902 (41.4)	10675 (44.1)	
Less than $20,000 per year	19412 (14.1)	6024 (24.9)	
Missing/don't know	9350 (6.8)	1524 (6.3)	
Education *(n, %)*			<.0001
Less than high school graduate	6244 (4.5)	2400 (9.9)	
High school graduate	23266 (16.9)	4358 (18.0)	
Some college	51386 (37.3)	9523 (39.3)	
College graduate	55702 (40.5)	7713 (31.9)	

Missing	1005 (0.7)	211 (0.9)	
CHD Risk Factors			
Diabetes *(n, %)*	6378 (4.6)	3548 (14.7)	<.0001
Hyperlipidemia *(n, %)*	18991 (13.8)	3664 (15.1)	<.0001
Hypertension *(n, %)*	44846 (32.6)	10005 (41.3)	<.0001
Cigarette smoking status *(n, %)*			<.0001
Never	68971 (50.8)	12459 (52.3)	
Past	57951 (42.7)	9159 (38.4)	
Current	8924 (6.6)	2218 (9.3)	
Family history of CHD *(n, %)*	16028 (11.7)	2376 (9.8)	<.0001
BMI *(kg/m^2) (mean, sd)*	27.8 (6.0)	29.7 (6.4)	<.0001
BMI categories *(n, %)*			<.0001
<25.0	50965 (37.1)	5644 (23.3)	
25-<30	47462 (34.5)	8484 (35.1)	
30-<35	24023 (17.5)	5850 (24.2)	
≥35	15022 (10.9)	4221 (17.4)	
Obese *(BMI≥30 kg/m^2) (n, %)*	39045 (28.4)	10071 (41.6)	<.0001
Physiological Measures *(mean, sd)*			
Systolic blood pressure *(mm Hg)*	127 (17.7)	130 (17.7)	<.0001
Diastolic blood pressure *(mm Hg)*	75 (9.2)	76 (9.4)	<.0001
Height *(cm)*	161.9 (6.6)	161.1 (6.7)	<.0001
Lifestyle Factors			
Physical activity *(MET-hours/week) (mean, sd)*	12.8 (13.8)	10.6 (13.1)	<.0001
Alcohol *(servings/week) (mean, sd)*	2.45 (4.87)	1.83 (4.87)	<.0001
Coffee drinker *(n, %)*	99066 (72.5)	16635 (69.3)	<.0001
AHEI 2010 *(mean, sd)*	52.4 (10.4)	49.6 (10.0)	<.0001

Calibrated total energy *(calories/day) (mean, sd)*	2298 (268.9)	2302 (284.0)	0.062
Care Issues *(n, %)*			
Insurance	130788 (95.9)	22019 (92.4)	<.0001
Regular healthcare provider	128538 (94.3)	21700 (91.0)	<.0001
Medications *(n, %)*			
Aspirin	29616 (21.5)	4683 (19.4)	<.0001
Diuretics	18217 (13.2)	4301 (17.8)	<.0001
Antihypertensive meds	14276 (10.4)	3171 (13.1)	<.0001
ACE Inhibitor	10649 (7.7)	2294 (9.5)	<.0001
ARB	977 (0.7)	159 (0.7)	0.3613
Beta blocker	11620 (8.4)	1930 (8.0)	0.0147
Ca channel blocker	12563 (9.1)	3194 (13.2)	<.0001
Lipid-lowering meds	11776 (8.6)	2121 (8.8)	0.2946
Multivitamin	55265 (40.2)	7789 (32.2)	<.0001
Hormone treatment			<.0001
Never	41367 (31.0)	11277 (47.7)	
Past	29056 (21.8)	7449 (31.5)	
Current	63133 (47.3)	4895 (20.7)	
Medical History at Baseline *(n, %)*			
CHD	4086 (3.0)	975 (4.0)	<.0001
COPD	4752 (3.7)	907 (4.0)	0.009
Atrial fibrillation	6054 (4.5)	1016 (4.3)	0.2042
Anemia (Hb<11 gm/dL)	927 (0.7)	299 (1.2)	<.0001
Hysterectomy	56599 (41.2)	11087 (45.8)	<.0001
Number of co-morbid conditions			<.0001
0	81041 (58.9)	14173 (58.6)	
1	30662 (22.3)	5924 (24.5)	

2+	25900 (18.8)	4108 (17.0)	
***WHI Group Membership** (n, %)*			
Study Arm			<.0001
Observational arm	87590 (63.7)	6086 (25.1)	
Clinical trial arm	50013 (36.4)	18119 (74.9)	
Hormone trial participant	13047 (9.5)	14300 (59.1)	<.0001
DM trial participant	40912 (29.7)	7923 (32.7)	<.0001
CAD participant	25981 (18.9)	10301 (42.6)	<.0001
EXT 1 participant	95556 (69.4)	19851 (82.0)	<.0001
EXT 2 participant	78499 (57.1)	15068 (62.3)	<.0001
MRC EXT 2 participant	7486 (5.4)	14830 (61.3)	<.0001
SRC EXT 2 participant	71013 (51.6)	238 (1.0)	<.0001
DbGAP consent			<.0001
No DbGap Consent	18590 (13.5)	5 (0.0)	
General Research Use	96433 (70.1)	21242 (87.8)	
Non Profit Use Only	22580 (16.4)	2958 (12.2)	
BMD participant	8876 (6.5)	2144 (8.9)	<.0001
SHARe	177 (0.1)	11831 (48.9)	<.0001
UNC HF cohort	20444 (14.9)	23730 (98.0)	<.0001

(Abbreviations: ACE = angiotensin-converting enzyme , ARB = angiotensin II receptor blocker, AHEI = alternative healthy eating index, BMD = bone mineral density, BMI = body mass index, Ca = calcium, CHD = coronary heart disease, COPD = chronic obstructive pulmonary disease, CVD = cardiovascular disease, DbGAP = database of genotypes and phenotypes, DM = dietary modification, EXT = extension, Hb = hemoglobin, MET = metabolic equivalent, MRC = medical records cohort, SES = Socioeconomic status, SRC = self-report cohort, UNC = University of North Carolina)

Table S2. Baseline characteristics stratified by heart failure

	No HF	*Any HF*		*HF-PEF (EF≥50%)*	*HF-REF (EF<40%)*	*HF-MEF (EF 40-49%)*
	n=21,097	n=2,212	***p-value***	n=1113	n=511	n=226
Demographics						
**Age (years) *(mean, sd)***	63.7 (7.3)	67.1 (6.6)	<0.001	67.1 (6.4)	66.1 (7.1)	67.5 (6.2)
**Age class *(n, %)***			<0.001			
**50-59 years**	6578 (31.2)	330 (14.9)		165 (14.8)	96 (18.8)	24 (10.6)
**60-69 years**	9620 (45.6)	1026 (46.4)		519 (46.6)	240 (47.0)	116 (51.3)
**≥70 years**	4899 (23.2)	856 (38.7)		429 (38.5)	175 (34.3)	86 (38.1)
**Race *(n, %)***			<0.001			
**Caucasian**	9887 (47.0)	1424 (64.5)		726 (65.4)	304 (59.5)	161 (71.6)
**African American**	7403 (35.2)	604 (27.4)		291 (26.2)	169 (33.1)	44 (19.6)
**Hispanic**	3430 (16.3)	141 (6.4)		77 (6.9)	31 (6.1)	13 (5.8)
**Other**	320 (1.5)	39 (1.8)		16 (1.4)	7 (1.4)	7 (3.1)
**Marital status *(n, %)***			0.001			
**Married or partnered**	11213 (53.5)	1094 (49.7)		548 (49.4)	257 (50.7)	114 (50.9)
SES						
**Income *(n, %)***			<0.001			
**$50,000 or greater**	5426 (25.7)	397 (18.0)		206 (18.5)	88 (17.2)	47 (20.8)
**$20,000 - <$50,000**	9275 (44.0)	1073 (48.5)		542 (48.7)	251 (49.1)	102 (45.1)
**Less than $20,000 per year**	5057 (24.0)	619 (28.0)		308 (27.7)	136 (26.6)	65 (28.8)
**Missing/don't know**	1339 (6.4)	123 (5.6)		57 (5.1)	36 (7.1)	12 (5.3)
**Education *(n, %)***			0.001			
**Less than high school graduate**	2035 (9.7)	192 (8.7)		97 (8.7)	44 (8.6)	21 (9.3)
**High school graduate**	3756 (17.8)	446 (20.2)		229 (20.6)	106 (20.7)	41 (18.1)
**Some college**	8238 (39.1)	913 (41.3)		445 (40.0)	211 (41.3)	102 (45.1)
**College graduate**	6883 (32.6)	643 (29.1)		337 (30.3)	145 (28.4)	57 (25.2)
**Missing**	185 (0.9)	18 (0.8)		5 (0.5)	5 (1.0)	5 (2.2)

CHD Risk Factors						
**Diabetes *(n, %)***	2814 (13.4)	502 (22.7)	<0.001	239 (21.5)	122 (23.9)	53 (23.5)
**Hyperlipidemia *(n, %)***	3075 (14.6)	387 (17.5)	<0.001	196 (17.6)	81 (15.9)	37 (16.4)
**Hypertension *(n, %)***	8289 (39.3)	1203 (54.4)	<0.001	593 (53.3)	279 (54.6)	118 (52.2)
**Cigarette smoking status *(n, %)***			<0.001			
**Never**	10994 (52.9)	1032 (47.4)		503 (45.7)	245 (48.8)	114 (51.6)
**Past**	7889 (38.0)	907 (41.6)		478 (43.4)	197 (39.2)	84 (38.0)
**Current**	1894 (9.1)	239 (11.0)		120 (10.9)	60 (12.0)	23 (10.4)
**Family history of CHD (n, %)**	1980 (9.4)	274 (12.4)	<0.001	135 (12.1)	74 (14.5)	26 (11.5)
**BMI (kg/m^2) *(mean, sd)***	29.5 (6.3)	30.9 (6.6)	<0.001	31.3 (6.7)	29.7 (5.8)	29.8 (6.3)
**BMI categories *(n, %)***			<0.001			
**<25.0**	5037 (23.9)	419 (19.0)		188 (16.9)	114 (22.4)	62 (27.4)
**25-<30**	7553 (35.8)	681 (30.8)		332 (29.8)	171 (33.5)	67 (29.7)
**30-<35**	5029 (23.8)	593 (26.8)		307 (27.6)	132 (25.9)	50 (22.1)
**≥35**	3474 (16.5)	518 (23.4)		286 (25.7)	93 (18.2)	47 (20.8)
**Obese (BMI≥30 kg/m^2) *(n, %)***	8503 (40.3)	1111 (50.3)	<0.001	593 (53.3)	225 (44.1)	97 (42.9)
Physiological Measures (mean, sd)						
**Systolic blood pressure (mm Hg)**	130 (17.5)	136 (18.5)	<0.001	136 (18.8)	135 (17.2)	137 (18.8)
**Diastolic blood pressure (mm Hg)**	76 (9.3)	76 (9.8)	0.441	76 (9.8)	77 (9.3)	78 (10.2)
**Height (cm)**	161.1 (6.7)	161.2 (6.5)	0.463	161.2 (6.4)	161.4 (7.2)	160.9 (6.4)
Lifestyle Factors						
**Physical activity (MET-hours/week) *(mean, sd)***	10.71 (13.09)	10.05 (12.62)	0.029	9.96 (12.81)	10.20 (12.45)	9.24 (11.25)
**Alcohol (servings/week) *(mean, sd)***	1.85 (4.81)	1.91 (5.43)	0.546	1.91 (5.64)	1.78 (4.29)	2.23 (5.39)
**Coffee drinker *(n, %)***	14462 (69.1)	1586 (72.2)	0.003	796 (72.0)	370 (73.1)	173 (77.2)
**AHEI 2010 *(mean, sd)***	49.7 (10.0)	49.4 (10.0)	0.196	49.3 (10.2)	49.2 (9.9)	49.8 (9.2)

**Calibrated total energy (calories/day)** ***(mean, sd)***	2301 (281.1)	2287 (291.4)	0.024	2299 (296.9)	2269 (272.9)	2251 (271.3)
**Walking limitation - 1 block** ***(n, %)***			<0.001			
**Yes, limited a lot**	434 (2.1)	83 (3.8)		45 (4.1)	14 (2.8)	9 (4.0)
**Yes, limited a little**	1682 (8.1)	259 (11.8)		147 (13.3)	54 (10.7)	20 (8.9)
**No limitation**	18697 (89.8)	1850 (84.4)		910 (82.6)	437 (86.5)	197 (87.2)
**SF-36 Fatigue** ***(mean, sd)***	63.4 (19.1)	60.9 (19.1)	<0.001	60.1 (19.0)	61.9 (19.0)	62.4 (19.2)
**SF-36 Physical Functioning** ***(mean, sd)***	79.3 (20.9)	71.8 (23.3)	<0.001	70.7 (23.6)	74.4 (22.2)	73.1 (23.0)
Care Issues (n, %)						
**Insurance**	19123 (92.1)	2075 (94.8)	<0.001	1044 (94.7)	477 (94.6)	211 (94.6)
**Regular healthcare provider**	18809 (90.6)	2070 (94.5)	<0.001	1047 (94.8)	480 (94.3)	202 (91.4)
Medications (n, %)						
**Aspirin**	3870 (18.3)	580 (26.2)	<0.001	284 (25.5)	134 (26.2)	57 (25.2)
**Diuretics**	3423 (16.2)	568 (25.7)	<0.001	290 (26.1)	119 (23.3)	50 (22.1)
**Antihypertensive meds**	2500 (11.9)	455 (20.6)	<0.001	221 (19.9)	109 (21.3)	41 (18.1)
**ACE inhibitor**	1782 (8.5)	344 (15.6)	<0.001	161 (14.5)	84 (16.4)	33 (14.6)
**ARB**	129 (0.6)	20 (0.9)	0.100	9 (0.8)	5 (1.0)	2 (0.9)
**Beta blocker**	1560 (7.4)	273 (12.3)	<0.001	149 (13.4)	50 (9.8)	24 (10.6)
**Ca channel blocker**	2547 (12.1)	443 (20.0)	<0.001	222 (20.0)	113 (22.1)	38 (16.8)
**Lipid-lowering meds**	1749 (8.3)	252 (11.4)	<0.001	128 (11.5)	53 (10.4)	26 (11.5)
**Multivitamin**	6788 (32.2)	744 (33.6)	0.163	387 (34.8)	160 (31.3)	81 (35.8)
**Hormone treatment**			<0.001			
**Never**	9803 (47.6)	1117 (52.1)		568 (52.1)	254 (51.4)	108 (49.8)
**Past**	6446 (31.3)	760 (35.4)		388 (35.6)	177 (35.8)	83 (38.3)
**Current**	4348 (21.1)	269 (12.5)		135 (12.4)	63 (12.8)	26 (12.0)
Medical History at Baseline (n, %)						
**CHD**	583 (2.8)	188 (8.5)	<0.001	79 (7.1)	58 (11.4)	18 (8.0)
**COPD**	725 (3.7)	108 (5.3)	<0.001	55 (5.3)	18 (3.8)	6 (2.9)

**Atrial fibrillation**	764 (3.7)	146 (6.8)	<0.001	68 (6.3)	31 (6.2)	12 (5.5)
**Anemia (Hb<11 gm/dL)**	234 (1.1)	33 (1.5)	0.111	16 (1.5)	9 (1.8)	4 (1.8)
**Hysterectomy**	9523 (45.2)	1071 (48.4)	0.003	527 (47.4)	248 (48.5)	119 (52.7)
**Dialysis history during follow-up**	22 (0.1)	64 (2.9)	<0.001	39 (3.5)	10 (2.0)	3 (1.3)
**Number of co-morbid conditions**			<0.001			
**0**	12939 (61.3)	1034 (46.8)		544 (48.9)	236 (46.2)	109 (48.2)
**1**	5101 (24.2)	660 (29.8)		321 (28.8)	142 (27.8)	82 (36.3)
**2+**	3057 (14.5)	518 (23.4)		248 (22.3)	133 (26.0)	35 (15.5)
Exposures						
**Serum creatinine (mg/dL)** ***(mean, sd)***	0.760 (0.178)	0.784 (0.210)		0.784 (0.196)	0.776 (0.197)	0.750 (0.166)
**Estimated GFR** ***(mean, sd)***	87.9 (16.1)	83.0 (16.9)		82.6 (16.4)	84.6 (17.8)	84.5 (15.1)
**CKD function group** ***(n, %)***			<0.001			
**Level 1 (eGFR<45)**	168 (0.8)	56 (2.5)		26 (2.3)	11 (2.2)	3 (1.3)
**Level 2 (45≤ eGFR <60)**	915 (4.3)	150 (6.8)		78 (7.0)	34 (6.7)	10 (4.4)
**Level 3 (60≤ eGFR <90)**	9877 (46.8)	1191 (53.8)		614 (55.2)	252 (49.3)	127 (56.2)
**Level 4 (90≤ eGFR ≤120)**	9889 (46.9)	797 (36.0)		388 (34.9)	205 (40.1)	85 (37.6)
**Level 5 (eGFR>120)**	248 (1.2)	18 (0.8)		7 (0.6)	9 (1.8)	1 (0.4)
Exposures (version 2)						
**Serum creatinine (mg/dL)** ***(mean, sd)***	0.760 (0.178)	0.784 (0.210)		0.784 (0.196)	0.776 (0.197)	0.750 (0.166)
**Estimated GFR** ***(mean, sd)***	87.3 (15.0)	83.4 (16.2)		83.5 (15.8)	84.4 (16.4)	86.0 (14.2)
**CKD 2021 function group** ***(n, %)***			<0.001			
**Level 1 (eGFR<45)**	198 (0.9)	58 (2.6)		30 (2.7)	10 (2.0)	3 (1.3)
**Level 2 (45≤ eGFR <60)**	906 (4.3)	142 (6.4)		67 (6.0)	39 (7.6)	7 (3.1)
**Level 3 (60≤ eGFR <90)**	9172 (43.5)	1019 (46.1)		522 (46.9)	229 (44.8)	105 (46.5)
**Level 4 (eGFR ≥ 90)**	10821 (51.3)	993 (44.9)		494 (44.4)	233 (45.6)	111 (49.1)

(Abbreviations: ACE = angiotensin-converting enzyme , ARB = angiotensin II receptor blocker, AHEI = alternative healthy eating index, BMI = body mass index, CHD = coronary heart disease, CKD = chronic kidney disease, cm = centimeter, COPD = chronic obstructive pulmonary disease, eGFR = estimated glomerular filtration rate, HF = heart failure, Hb= hemoglobin, SD = standard deviation, SES = Socioeconomic status)

Table S3. Prespecified subgroup analysis for association of eGFR with any heart failure (eGFR ≥90 = reference level)

**Model**	n	# events	**HR (95% CI)** *per 10 eGFR unit decrease*	***eGFR<45*** HR (95% CI)	***45≤eGFR<60*** HR (95% CI)	***60≤eGF<90*** HR (95% CI)	***p-trend***
No Diabetes							
**Fully Adjusted***	19,974	1,517	1.08 (1.06, 1.09)	3.14 (2.72, 3.62)	1.26 (1.15, 1.38)	1.03 (0.99, 1.08)	<0.001
With Diabetes							
**Fully Adjusted***	3,316	447	1.13 (1.10, 1.16)	2.31 (1.83, 2.91)	2.04 (1.77, 2.35)	1.09 (1.00, 1.18)	<0.001
No HTN							
**Fully Adjusted***	13,817	881	1.07 (1.05, 1.09)	2.70 (1.91, 3.83)	1.75 (1.55, 1.98)	1.00 (0.95, 1.05)	<0.001
With HTN							
**Fully Adjusted***	9,492	1,083	1.08 (1.06, 1.09)	2.62 (2.30, 2.99)	1.24 (1.13, 1.37)	1.04 (0.99, 1.09)	<0.001
No CHD history							
**Fully Adjusted***	22,538	1,799	1.08 (1.07, 1.10)	2.54 (2.24, 2.89)	1.42 (1.31, 1.54)	1.01 (0.98, 1.05)	<0.001
Prevalent CHD							
**Fully Adjusted***	771	165	1.05 (1.01, 1.09)	2.53 (1.68, 3.79)	1.66 (1.34, 2.07)	1.19 (1.05, 1.36)	<0.001
Never used hormone treatment							
**Fully Adjusted***	10,920	1,023	1.09 (1.07, 1.11)	2.49 (2.10, 2.96)	1.45 (1.31, 1.62)	1.01 (0.96, 1.06)	<0.001
Past hormone treatment							
**Fully Adjusted***	7,206	682	1.08 (1.06, 1.10)	2.73 (2.27, 3.27)	1.47 (1.31, 1.66)	1.08 (1.01, 1.14)	<0.001
Current hormone treatment							
**Fully Adjusted***	4,617	248	0.95 (0.91, 1.00)	1.75 (0.97, 3.16)	0.56 (0.40, 0.78)	0.91 (0.81, 1.03)	0.016
White, non-Hispanic							
**Fully Adjusted***	11,311	1,258	1.07 (1.05, 1.08)	2.51 (2.20, 2.86)	1.34 (1.24, 1.46)	1.01 (0.97, 1.05)	<0.001
Black/African American							
**Fully Adjusted***	8,007	542	1.11 (1.07, 1.15)	2.24 (1.60, 3.14)	1.57 (1.23, 2.00)	1.18 (1.01, 1.37)	<0.001
Hispanic							
**Fully Adjusted***	3,571	128	1.13 (1.03, 1.24)	2.58 (0.87, 7.69)	2.00 (1.09, 3.66)	1.11 (0.81, 1.52)	0.023
Age < 65 years							

**Fully Adjusted***	11,303	572	1.09 (1.07, 1.12)	3.15 (2.30, 4.32)	2.93 (2.53, 3.39)	0.91 (0.84, 0.98)	<0.001
Age ≥ 65 years							
**Fully Adjusted***	12,006	1,392	1.08 (1.06, 1.09)	2.60 (2.28, 2.97)	1.17 (1.07, 1.28)	1.05 (1.01, 1.10)	<0.001

*Adjusted for age, race, ethnicity, marital status, income, education, diabetes mellitus, hypertension, systolic blood pressure, diastolic blood pressure, atrial fibrillation, hysterectomy, history of coronary heart disease, body mass index, physical activity, smoking status, diet quality, coffee intake, alcohol intake, ability to walk 1 block. (Abbreviations: CHD = coronary heart disease, HTN = hypertension)
